# Human development and entrepreneurship: A cross-country analysis of early-stage, intention, and discontinuation

**DOI:** 10.1371/journal.pone.0313678

**Published:** 2024-11-25

**Authors:** Cristian A. Rojas, Felipe Chávez-Bustamante, Rolando Rubilar-Torrealba

**Affiliations:** 1 Departamento de Ciencias Económicas y Administrativas, Universidad Católica de Temuco, Temuco, Chile; 2 Center of Applied Ecology and Sustainability (CAPES), Santiago, Chile; 3 Facultad de Economía y Gobierno, Universidad San Sebastián, Valdivia, Chile; 4 Departamento de Industrias, Universidad Técnica Federico Santa María, Valparaíso, Chile; Sapienza University of Rome, ITALY

## Abstract

Entrepreneurship is widely recognized as a vital driver of economic growth, yet its relationship through different stages with broader, non-purely monetary dimensions still needs to be explored. This research seeks to fill this gap by investigating the association between human development and entrepreneurship, recognizing the crucial role of environmental conditions in shaping entrepreneurial activity. Our cross-country Bayesian analysis shows strong evidence that a nation’s level of human development is associated with entrepreneurial activity in three stages: early entrepreneurship, intention for future engagement, and discontinuation of ventures. Our findings expand the intricate relationship between entrepreneurship and economic variables, highlighting the importance of environmental conditions in shaping entrepreneurial behavior. This article emphasizes that promoting robust entrepreneurial ecosystems requires considering integral dimensions such as human development. Policymakers and researchers should broaden their perspectives to encompass these holistic dimensions to create an environment conducive to entrepreneurial growth.

## Introduction

Entrepreneurship has long been deemed an essential promoter of economic growth and development. Since Baumol [1], a substantial body of literature has highlighted the essential role of entrepreneurs in economic development [2–4]. Entrepreneurship contributes to increased employment rates, increased productivity, and GDP growth [5] through its role in market innovation, the promotion of new ventures, and the capitalization of business opportunities and knowledge spillovers [6–10].

While there is a consensus on the critical role of entrepreneurship in promoting economic growth [11], the reciprocal relationship between economic conditions and entrepreneurial activity has received comparatively less attention in the academic literature. Previous studies looking at feedback between economic growth, innovation, and entrepreneurship found a positive reinforcing relationship, with entrepreneurship generating economic growth and higher economic growth promoting entrepreneurship [2, 6, 12, 13]. The latter phenomenon can be explained by recognizing that better economic conditions generate new opportunities, making it easier for entrepreneurs to find them and exploit them [14].

A growing branch of literature that emphasizes the importance of contextual effects in shaping entrepreneurial activity is the entrepreneurial ecosystem approach [5]. It focuses on the interdependence of elements in the entrepreneurial landscape [15], illustrating the profound influence of environmental conditions on entrepreneurial outcomes. These conditions, often represented in the health and dynamism of the entrepreneurial ecosystem, constitute the background where entrepreneurship grows. Despite growing interest in this ecosystem approach, it is not well understood how agents, upon setting up their ventures, adapt to environmental changes and—or—shape the environment [16].

Just as ecosystems depict certain facets of a country’s environment, the concept of human development offers another lens to examine these contextual conditions. Human development—measured by the HDI through education, health, and income indicators—can influence and be influenced by economic growth and entrepreneurial activity [17]. Entrepreneurship may translate into not only GDP growth, but also more complex social dimensions such as human development [17, 18]. This idea is also related to viewing entrepreneurship as the ‘freedom to do as one wants’—the Capability Approach—suggesting the environment creates the freedom for entrepreneurs to pursue their ventures [19]. Consequently, a careful understanding of human development as an indicator of environmental conditions in a country may enrich our understanding of contextual factors that drive entrepreneurial results.

Human development emerged as a response to the narrow focus of economic growth as a society’s target, arguing that it neglects other social dimensions [20]. With the premise that human development plays a role in expanding people’s elections—with the most important being related to living a long healthy life, acquiring education, and accessing resources for a decent living—the United Nations Development Programme (UNDP) proposed the Human Development Index (HDI) [21, p.9]. The HDI is a composite index computed as a geometric mean of the country-level dimensions of education, health, and income, providing a more holistic understanding of the development of nations, in contrast to other standard GDP measures. It has been claimed that the proper gauge of development or progress should be human development growth, indicated by HDI dimensions, rather than merely focusing on economic expansion [22].

Despite the shift from GDP-based analysis to more comprehensive development measures, the connection between entrepreneurship and these measures remains relatively unexplored in the existing literature. Most studies have focused on the impact of entrepreneurship on human development [23], stating that innovation and creativity, derived from entrepreneurial activities, improve quality of life [24, 25]. As a result, public policy often aims to promote entrepreneurship as a means to promote human development and stimulate economic growth [13]. Recognizing the necessity of considering noneconomic factors, it becomes crucial for entrepreneurship researchers to avoid limiting their analyzes to only economic or monetary indicators [26], as it can lead to an incomplete understanding of the entrepreneurial landscape. Furthermore, we leverage the idea that a country’s HDI reflects environmental conditions that could impact entrepreneurial activity through its components: education, healthcare, and economic resources. Hence, we advance the view that it is only economic growth that reinforces entrepreneurship by also complementing it with the idea that the education and healthcare systems—a more integral view of development—could be playing a relevant role as well. This argument is rooted in the idea that education and health, have been found to be relevant to the entrepreneurship phenomenon (see, for example, [27–30]).

Recognizing the multifaceted nature of entrepreneurship—influenced by economic and noneconomic factors—we study whether interacting contextual factors (related to human development indicators) are associated with entrepreneurial activity. We address the following questions: Does a country’s level of human development relate to its entrepreneurial activity? Does this relationship vary according to the stage of entrepreneurial activity? To answer them, we collected country-level data from 2010 to 2018 from the Global Entrepreneurship Monitor (GEM)—entrepreneurial activity and other country-level perception-related variables—and from the Human Development Report (HDR)—the HDI—and analyzed it using Bayesian statistics.

This research empirically studies the dynamic relationship between human development and entrepreneurial activity in various countries over time, considering three stages of entrepreneurial activity: early entrepreneurship, entrepreneurship intention, and entrepreneurial discontinuance. Our contribution to the literature is twofold. First, we contribute to the discussion of the interconnection between growth, entrepreneurship, and the role of the latter in the progress of a country [31]. As an intricate network is formed between these variables, it becomes essential to comprehend them to gain insight into how to foster robust entrepreneurial ecosystems. As argued by Gries and Naudé [18], entrepreneurship should be viewed as a policy objective on its own, calling for a shift in the focus from entrepreneurship toward economic growth to viewing growth—and hence, improved opportunities in the environment—as fertile grounds for promoting entrepreneurship. In this regard, we particularly expand the view of human development and entrepreneurship by analyzing a direction of the dyad that has been mostly neglected: environmental macroeconomic conditions set the ground for entrepreneurship to unfold. Second, the article contributes to increasing the diversity of entrepreneurship studies by incorporating different entrepreneurial stages for multiple economies, since most existing studies have focused on nascent stages in developed economies [32]. While most of the related work on this topic has operationalized entrepreneurship by considering the Total Early Stage Entrepreneurship (TEA) rate and its variations (see for example [33, 34]), we chose to complement this view and consider the entrepreneurial phenomenon through three stages: ideation (intention), early entrepreneurship, and discontinuance. Through this, we can offer novel insights by building on the idea that entrepreneurship is not a static phenomenon, but rather a transitional one that requires navigating subsequent stages [35–37].

### Conceptual background

According to Edelman and Yil-Renko [38, p. 835], a challenge for empirical research is to “capture the effects of objective environment and entrepreneurial perceptions as the venture-creation process unfolds.” The authors called for a mixture of entrepreneurial perceptions and objective measures of environmental conditions. This view of the entrepreneurial process resembles the proposition of Davidsson [39] about reconceptualizing the opportunity concept in Shane and Venkataraman’s seminal work [40] through three constructs: ‘external enablers’—aggregate level circumstances—‘new venture ideas’—a mixture of not yet existent offerings, markets, and means to bring them to life—and ‘opportunity confidence’—perceived evaluation of how attractive a stimulus is. Taking these ideas into account, our approach to understanding the association between entrepreneurship and human development considers environmental conditions and individual perceptions. HDI—our operationalization of human development as the key study variable—objectively quantifies the conditions of a country’s environment in its three dimensions.

At the country level, there are several articles that study the determinants that influence entrepreneurial rates. Dvouletỳ [41] found that even when considering different measures of entrepreneurial activity, the unemployment rate and economic freedom positively affect entrepreneurial activity, while bureaucratic procedures negatively affect them. Alternatively, Rusu and Roman [42] highlighted that the key macroeconomic determinants of entrepreneurial activity were related to inflation, foreign direct investments, access to finance and taxation. Through a Bayesian model averaging approach, Arin et al. [43] showed that key variables in explaining aggregate entrepreneurial activity are GDP per capita, unemployment, marginal tax rates, and inflation volatility. Regarding sustainable entrepreneurship over time, Moya-Clemente et al. [44] found that environmental factors, such as the availability of water and sanitation, as well as land use (sustainable development goals 7 and 15, respectively) and economic factors, such as decent work, economic growth, infrastructure, and innovation (sustainable development goals 8 and 9), were key predictors of entrepreneurial continuation. In general, this line of research suggests a relationship between economic factors and entrepreneurial activity, with contextual factors providing conditions to improve entrepreneurial activity.

It is relevant to point out that there are many articles that study different determinants of entrepreneurial activity at the aggregate level, but there is no consensus on which particular factors improve it [42]. Trying to encompass every macroeconomic variable that has been previously used (e.g., economic freedom, taxation, bureaucracy, inflation rates, unemployment) would make the analysis too complex and would fall outside the scope of the article. Although we decided to focus on measurements of human development, HDI could capture other determinants indirectly.

#### Individual determinants of entrepreneurial activity

Following the eclectic theory of Verheul et al. [45], we considered not only the macroeconomic determinants of entrepreneurial activity, but also the microeconomic behavioral components related to the development of ventures.

The literature has studied how individual attitudes and behaviors directly affect entrepreneurial activity. For example, Kautonen et al. [46] tested the theory of planned behavior, finding that attitude, perceived behavioral control (PBC), and subjective norms are significant predictors of intentions to engage in entrepreneurship. The authors also found that both intention and PBC significantly predict subsequent engagement in entrepreneurial behavior. More recent studies have also found supporting evidence for the idea that attitudes toward entrepreneurship, as well as entrepreneurial intention, are positively associated with actual entrepreneurial behavior [47–49].

In general, we summarized the individual determinants of entrepreneurial activity related to the perception of people about the following variables: opportunity, skills, status, fear, and equality.

Opportunities have been at the core of the entrepreneurial literature [40]. The idea is that people become motivated by their perception of business opportunities, transform their intentions into actions, and create their own ventures [38], and it has been found that perception of opportunities was positively associated with entrepreneurial intention [50]. Furthermore, there is an established idea that opportunities need a certain degree of skills for entrepreneurs to exploit them, so academic research has shown that skill perception is positively associated with national rates of venture creation [51]. The belief in self-skills—also known as self-efficacy in the literature—has been found to be a key driver of entrepreneurial behavior [52]. Koellinger et al. [53] found that belief in self-skills is a key factor when starting a business, with earlier stages of entrepreneurship showing greater confidence than those who participated in ventures longer. Hsu et al. [52] argued that the effect of self-efficacy on entrepreneurial intention is mediated by the fit of the individual with the environment, finding that capable agents may be discouraged from intent to engage in a venture if they do not perceive that supplies of entrepreneurship can fulfill their necessities. In general, the findings of the literature mostly support the idea that perceptions of their own skills are associated with the intention to create new businesses [50].

Regarding the status attributed to entrepreneurs, Boldureanu et al. [54] showed that exposing entrepreneurs as role models (through teaching) becomes relevant in affecting the student’s entrepreneurial intentions and attitudes. It has also been stated in the literature that some entrepreneurs respond that other people affected their decision to engage in their ventures when asked why they did it, so role models have played a key role in the entrepreneurial decision process [55]. Other academic research has also found supporting evidence for the idea that the figure of a role model—which, in this paper, we operationalize as the status attributed to entrepreneurs given its likely role as an inspirator of entrepreneurship as a career option [56]—can become drivers of entrepreneurial activity [57–59].

Another key psychological issue affecting (potential) entrepreneurs is fear of failure in their ventures, which has been indicated as a deterrent for entrepreneurship [60, 61]. It may not only deter the intention of individuals to engage in entrepreneurship, but it may also affect those already involved in entrepreneurial ventures [62]. Furthermore, fear could dampen people’s assessment of their abilities and skills, so even if people crystallize objective capacities for entrepreneurship, they might not act on them [63].

Finally, entrepreneurship can serve as a way to reach a higher standard of living, especially in unequal societies where entrepreneurship can build a path to social mobility [64]. Bruton et al. [65] called for a deeper understanding of economic inequality in academic research related to entrepreneurship. Inequality, as well as other socioeconomic phenomena, could also have implications for entrepreneurship, as the entrepreneurial phenomenon has also been characterized by the potential to cause change in economic, social, institutional, and other dimensions [66].

#### Human development and entrepreneurial activity

Since the late 1990s, entrepreneurship has been recognized as a fundamental driver of economic growth due to the unique ability of the entrepreneur to convert knowledge into commercially viable and innovative activities [67]. However, the increase in productivity and economic growth driven by entrepreneurial innovation does not inherently guarantee elevated levels of human development [18]. Despite the importance of human development and its dimensions, academic research on its relationship with entrepreneurship has been limited [24–26].

A key study that drew on the ‘Capability Approach’ to emphasize the pivotal role of entrepreneurship in human development, both as a catalyst for expanding others’ capabilities and as a ‘valuable functioning’ in its own right, is that of Gries and Naudé [18]. Their work suggested that entrepreneurship transcends the traditional role of an economic driver and can be a goal in its own right, describing it as a human function that can even enhance human capabilities. However, the authors noted that entrepreneurship is not inherently effective and its worth can weaken when perceived as a required endeavor instead of a choice or when it is difficult to connect ideas with suitable opportunities. Building on that work, Ballesta et al. [24] studied whether innovative entrepreneurship contributions go beyond monetary profits. Their results indicated a positive association between innovative entrepreneurial activity and human development, a result attributed to the idea that entrepreneurship improves human capabilities and, with it, improves dimensions of human development.

Recently, Ibourk and Raoui [68] investigated the influence of collaborative entrepreneurial activities on human development in Morocco. They found that cooperative entrepreneurship played a key role in the adjustment of socio-spatial inequalities by increasing access to essential services (education, health, and employment) in regions characterized by suboptimal human development.

Rodríguez-Pose and Palavicini-Corona [69] investigated whether different elements of Mexico’s Local Economic Development had contributed to improve human development in counties. The study did not find sufficient statistical evidence to support the hypothesis that municipalities that promote entrepreneurship should experience greater levels of development. Similarly, Dvouletý et al. [23] did not demonstrate any influence of entrepreneurship on HDI. On the contrary, the study by Rani and Kumar [25] found a positive correlation between total entrepreneurial activities and HDI levels in BRICS economies, suggesting that policy makers should strengthen entrepreneurial ecosystems to improve human development.

As seen, within that narrow body of scholar work on human development and entrepreneurship, most of it has approached the relationship from a unique direction, as it is (allegedly) entrepreneurship that pushes higher levels of human development. However, the particular perspective of the dyad we aim to study is the other one: human development sets the environmental circumstances (from a monetary, educational and health perspective) that could condition entrepreneurship through different stages. Academic works that have studied the phenomenon in the same direction as we aim, is that of Amorós and Cristi (2011) and Maniyalath and Narendran (2016). First, Amorós and Cristi [17] studied the relationship between poverty and entrepreneurial activities, testing whether higher poverty levels and income inequality are related to higher entrepreneurial activity. Through pooled data analysis, they found a U-shaped curve between poverty and entrepreneurial activity (measured as early-stage entrepreneurial activity and necessity-based entrepreneurial activity). The authors argued that this is due to some entrepreneurs being attracted to engage in ventures for opportunity motives, while others are pushed out of necessity. Second, Maniyalath and Narendran [26] studied whether national-level characteristics—such as HDI, the gender inequality index, and national religious composition—explain female entrepreneurship rates. They found a positive association between HDI and female entrepreneurial activity, arguing that practices centered on human development pave the way for women to become entrepreneurs. Thus, considering this scarce literature, and also, the relevance that the dyad entrepreneurship-development implies for economies around the world, we aim to contribute to this gap by extending the idea that environmental conditions depicted by HDI at a national level is related to entrepreneurial activity through different stages.

Drawing on the literature above, the hypothesis of this work is as follows:

**Hypothesis 1**. *There is a positive association between higher levels of human development and entrepreneurial activity*.

## Materials and methods

We built an annual cross-country database for the years 2010 to 2018 using entrepreneurship-related information from the Global National Level Data database from the Adult Population Survey (APS) developed by the Global Entrepreneurship Monitor (GEM) [70] and HDI from the UN Human Development Reports [71]. The resulting database covers 58 countries (Table 1) in nine years.

**Table 1:** List of countries used in the study.

Countries			
Angola	Argentina	Australia	Barbados
Belgium	Botswana	Brazil	Bulgaria
Burkina Faso	Cameroon	Canada	Chile
China	Colombia	Croatia	Cyprus
Egypt	Estonia	Finland	France
Germany	Greece	Guatemala	Hungary
India	Indonesia	Ireland	Israel
Italy	Japan	Lithuania	Luxembourg
Madagascar	Malaysia	Mexico	Morocco
Netherlands	Norway	Panama	Peru
Philippines	Poland	Portugal	Qatar
Romania	Saudi Arabia	Singapore	Slovakia
Slovenia	South Africa	Spain	Sweden
Switzerland	Thailand	Turkey	United Arab Emirates
Uruguay	USA		

The dependent variables used to understand the different stages of entrepreneurship are the following (definitions available on the GEM website [72] and the database labels):

- Total early stage entrepreneurial activity (TEA): The proportion of individuals between 18 and 64 years of age who are budding entrepreneurs or owners-managers of a new venture.
- Entrepreneurial intention (Futsup): The proportion of people between 18 and 64 years of age who expect to start a new business in the next three years (excluding those already involved in entrepreneurship).
- Entrepreneurial discontinuance (Dissent): The proportion of individuals between 18 and 64 years of age who exited a business in the past year and the business did not continue.

HDI, our explanatory variable of interest, corresponds to a geometric mean of three dimensions: education, health, and income. The dimension of education is gauged through the expected and average years of schooling. Health is quantified through life expectancy at birth and income dimension is measured through gross national income (GNI) per capita in terms of purchasing power parity.

To control for specific entrepreneurial national-level aspects, we used the following variables (definitions available in the GEM website [72]:

- Status: The proportion of individuals aged between 18 and 64 years who agree with the statement that successful entrepreneurs receive high status in their country.
- Opportunities: The proportion of individuals aged between 18 and 64 years, excluding those involved in any stage of entrepreneurial activity, who see good opportunities to start a firm in the area where they live.
- Skills: The proportion of individuals aged between 18 and 64 years, excluding those involved in any stage of entrepreneurial activity, who believe they have the required skills and knowledge to start a business.
- Fear: The proportion of individuals aged between 18 and 64 years, excluding those involved in any stage of entrepreneurial activity, who point out that fear of failure would dissuade them from setting up a business.
- Equality: The proportion of people aged between 18 and 64 who agree that most people would prefer that everyone had a similar standard of living in their country.

To analyze the relationship between human development and the different stages of entrepreneurship, we examined the data with Bayesian mixed effect models using the Hamiltonian Monte Carlo algorithm implemented in Stan through the brms package [73] in the statistical software R (v4.2.3) [74] (another relevant article on entrepreneurship using Bayesian analysis is Arin et al. [43] and for other Bayesian applications on entrepreneurial decision making, see Lohrke et al. [75]).

Our three models (one for each stage of entrepreneurship studied) attempted to explain the relationship between human development and entrepreneurship while accounting for the influence of other factors and controls at the national level. Due to measurement lags in HDI, and since human development might not translate into entrepreneurship activity in the same year, we used lagged HDI as an independent variable; i.e., entrepreneurial activity in a current period (year *t*) relates to a country’s human development conditions in the previous period (year *t* − 1). All models used a Gaussian distribution for the dependent variables. The simulations used three chains of 8000 iterations with 2000 burn-ins, leaving 18000 samples in the posterior distribution of each parameter. Priors were assumed to follow a normal distribution with a mean of zero (weakly informative priors) for all coefficients in all models. The distributions of the different chains in the different models and the scale reduction factor (R-hat∼1) indicated convergence and stability in all models.

All models included national-level covariates of status, opportunities, skills, fear, and equality for the current period (year *t*) and the previous period (year *t* − 1) to account for potential lagged effects. To account for dynamic influences in the different entrepreneurial stages (dependent variable), we included their lagged value (year *t* − 1) as an independent variable. In addition, random effects by country (intercept and slope) and year (intercept) were incorporated into the regressions. This was done to control for the intrinsic differences and variability between countries over the years, which may directly influence our regression model. Furthermore, we allowed these random effects to affect the estimated covariates to accommodate unobserved country-specific heterogeneity. Fixed effects for each country were also included to capture persistent and non-transitory characteristics unique to each country, which might systematically affect our outcomes of interest. This approach allowed us to account for potential confounding factors that are particular to each country over time—including social, cultural, and institutional factors—which have not been included explicitly. All covariates were standardized (mean-centered and divided by standard deviation) to avoid scale issues and convergence problems during estimation. Alternative models (changing/excluding lags, covariates, or random effects) did not provide different qualitative results or significantly improved cross-validation measures (LOOIC or K-fold) and were worse at explaining extreme values of the entrepreneurship stages.

## Results

The different stages of entrepreneurship—early, intention, and discontinuance—were analyzed in three separate Bayesian mixed-effect models using the same covariates.

### Early entrepreneurship (TEA)

**Fig 1:**
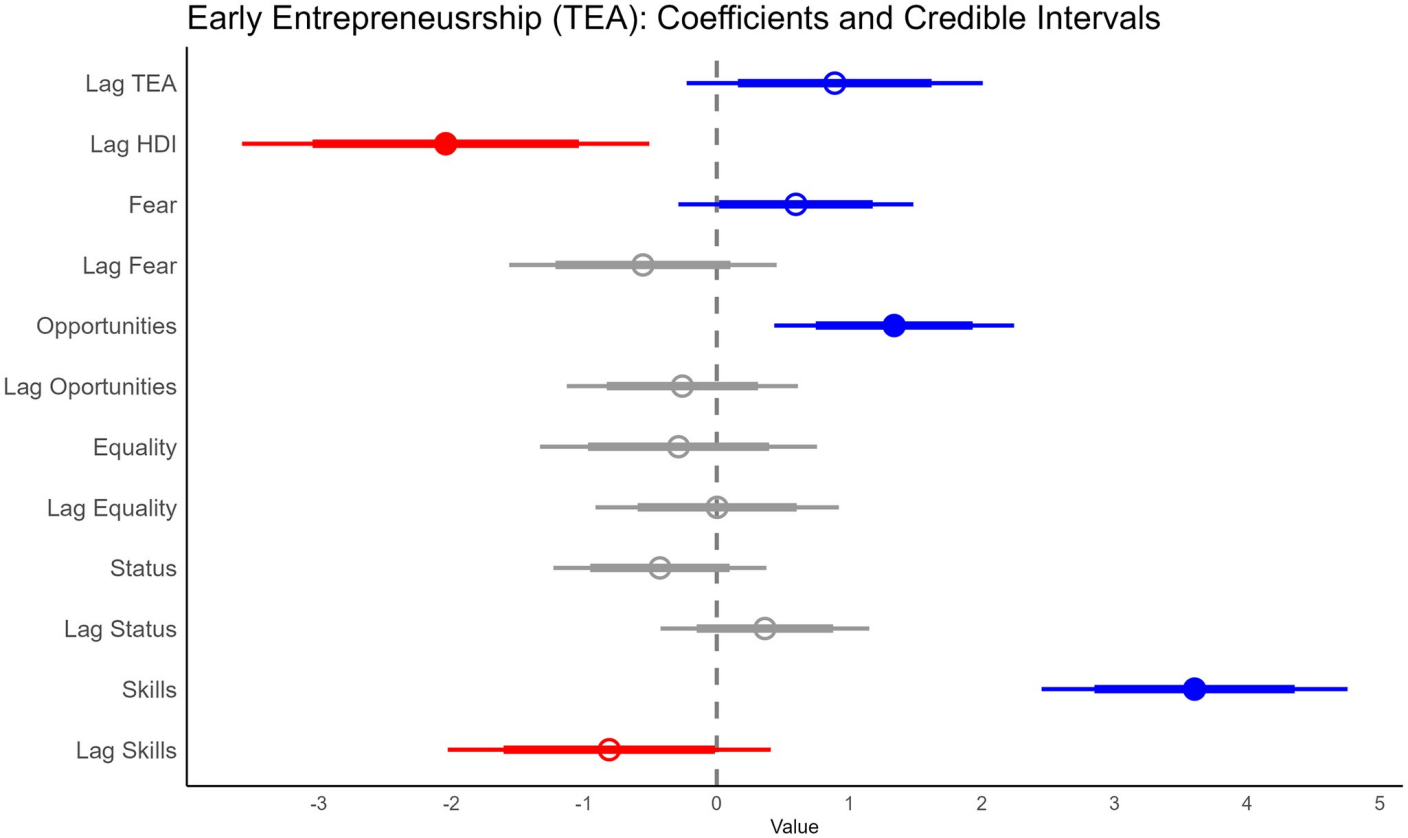
Coefficient plot for TEA. It shows how early entrepreneurship is related to its level in the previous year, to HDI, and to the proportion of people who fear failure, believe there are good opportunities to start a firm, desire equality in society, perceive entrepreneurs have high social status, and think they have skills to start a business in a given year. ‘Lag’ represents the previous year. The intercept is suppressed for simplicity. The thick lines represent an 80% CI, while the thin lines are 95% CI.

**Fig 2:**
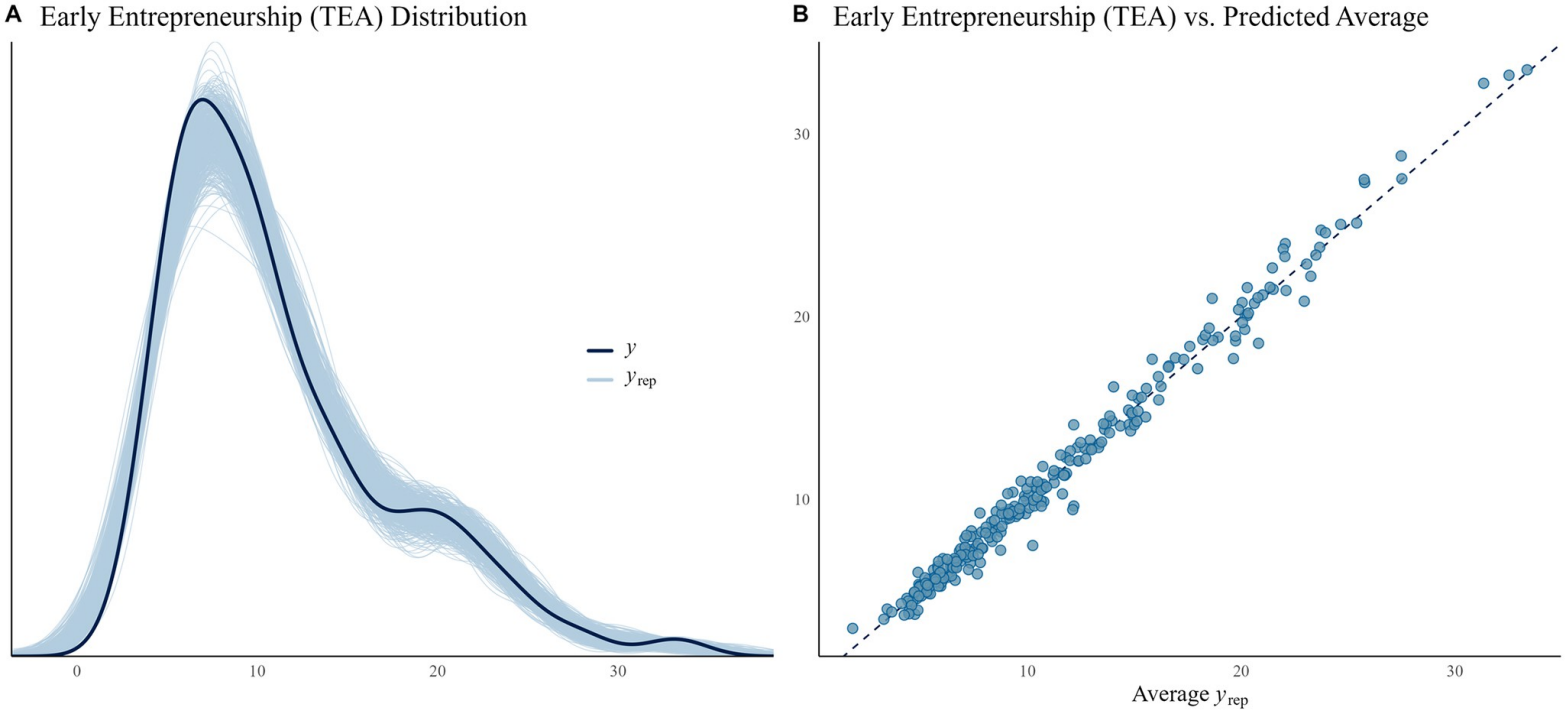
Model fitness of early entrepreneurship (TEA). A: Distribution (*y*) and random sample (500 randomly-selected replications) of predicted distributions (*y*_*rep*_). B: Average estimation of early entrepreneurship vs. actual early entrepreneurship.

For early entrepreneurship, the Bayesian model shows that HDI in the previous period has a negative relation to TEA (i.e., over 95% of the distribution of the estimated median is less than zero). As for other covariates, previous year early entrepreneurship, ‘Fear’, ‘Opportunities’, and ‘Skills’ have a positive relationship with early entrepreneurship (i.e., at least the 80% credible interval (CI) of their estimated median distribution was greater than zero); while the proportion of people believing that they had the required skills to start a business in the previous year (‘Lag Skills’) had a negative relationship (Fig 1). The other covariates do not seem to influence early entrepreneurship (their coefficients had an important part of their—estimated—distribution to both sides of the zero threshold). Fig 2 illustrates how closely the model can replicate early entrepreneurship, contrasting the data with the model’s estimated median; panel A shows the distribution, while panel B shows the average.

Figures are provided for all three models. They graphically illustrate how close the simulations can replicate the actual distribution of the data, i.e., the fit of the model. While the left panel depicts the actual distribution, the right panel considers the average of the prediction. Each blue line represents a randomly chosen distribution simulation, while each blue dot represents the predicted average. The better the fit, the closer the simulations are grouped around the lines (the continuous line in the left panel and the dashed one in the right panel).

### Entrepreneurial intention (Futsup)

**Fig 3:**
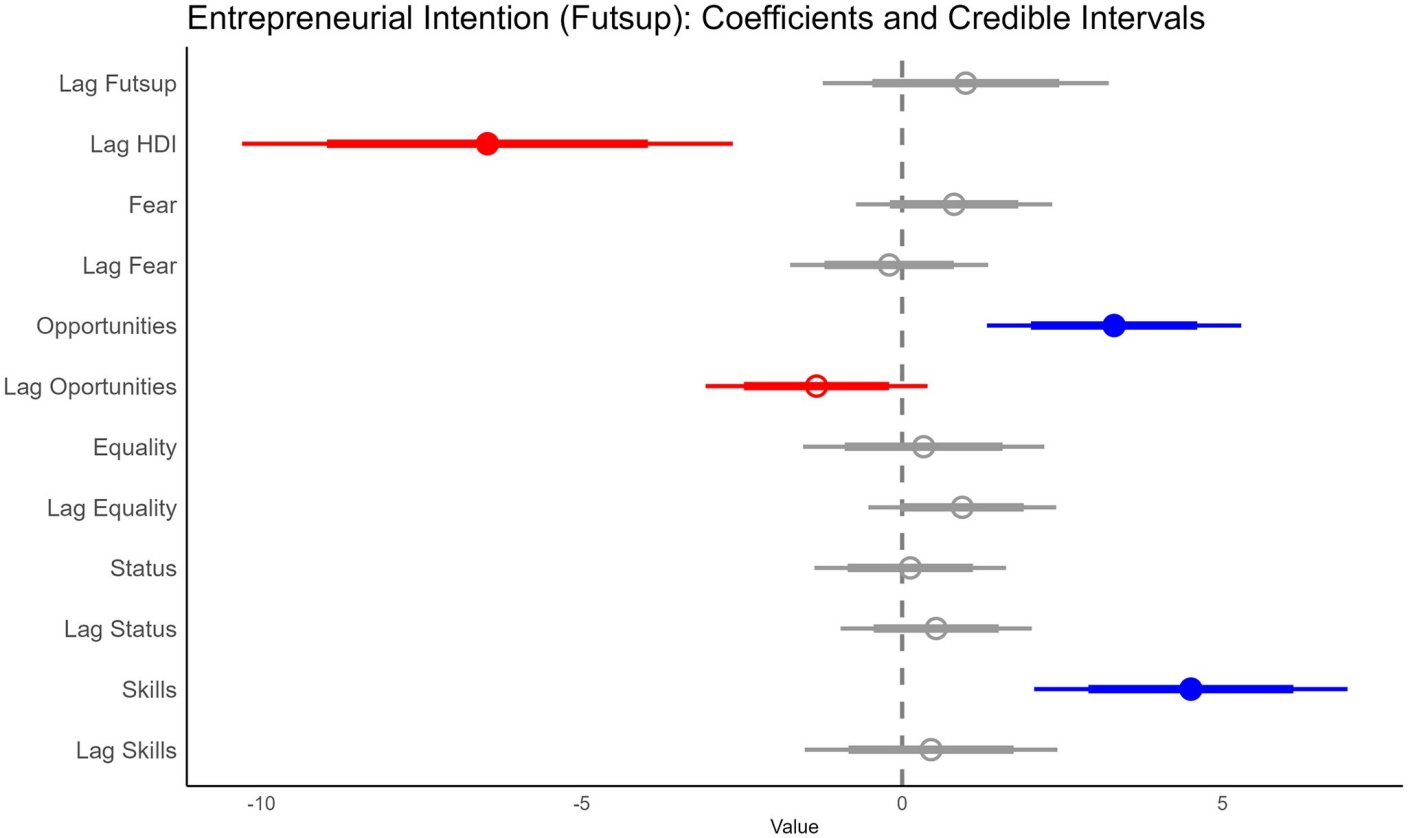
Coefficient plot for Futsup. It shows that intention is related to its level in the previous year, to HDI, and to the proportion of people who fear failure, believe there are good opportunities to start a firm, desire equality in society, perceive entrepreneurs have high social status, and think they have skills to start a business in a given year. ‘Lag’ represents the previous year. Intercept is suppressed for simplicity. Thick lines represent an 80% CI, while thin lines are 95% CI.

**Fig 4:**
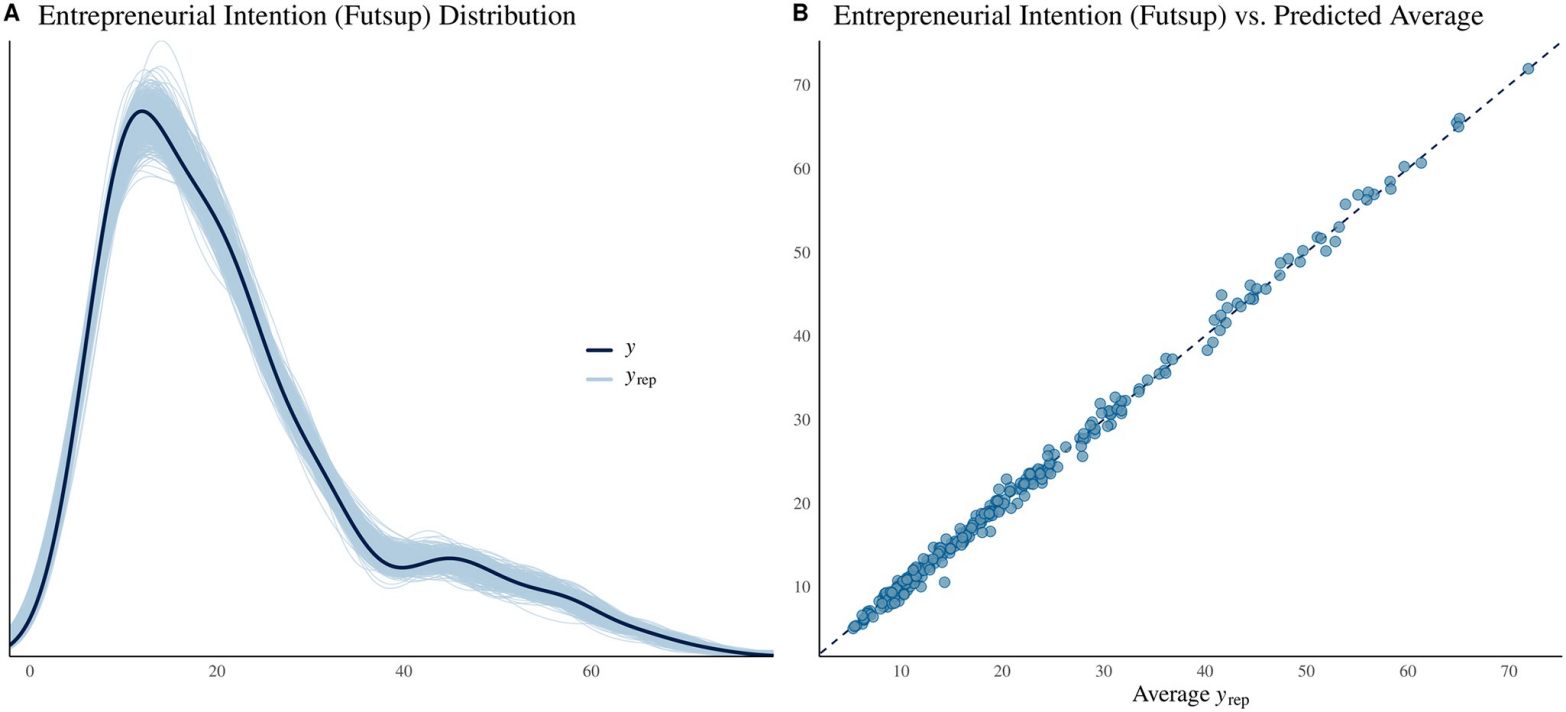
Model fitness of entrepreneurship intention (Futsup). A: Distribution (*y*) and random sample (500 randomly-selected replications) of predicted distributions (*y*_*rep*_). B: Average estimation of entrepreneurship intention vs. actual entrepreneurship intention.

For entrepreneurial intentions, the results are similar to those of early entrepreneurship. HDI in the previous period has a negative relation (over 95% of the distribution of the estimated median is less than zero) to ‘Futsup’, while ‘Opportunities’ and ‘Skills’ have a positive relation to it (the 95% CI of their estimated median distribution is greater than zero). ‘Lag Opportunities’ seems to have a negative relation to entrepreneurial intention, with the 80% CI of its estimated median distribution being less than zero. The relation of other covariates to the intention of entrepreneurship cannot always be determined to be different from zero (that is, at least 20% of the distribution of their estimated median is above and below zero; see Fig 3). Fig 4 shows how closely the model is able to replicate the entrepreneurial intention; panel A shows the estimated distribution and the actual distribution of Futsup, while panel B shows the average estimation versus the data.

### Entrepreneurial discontinuance (Dissent)

**Fig 5:**
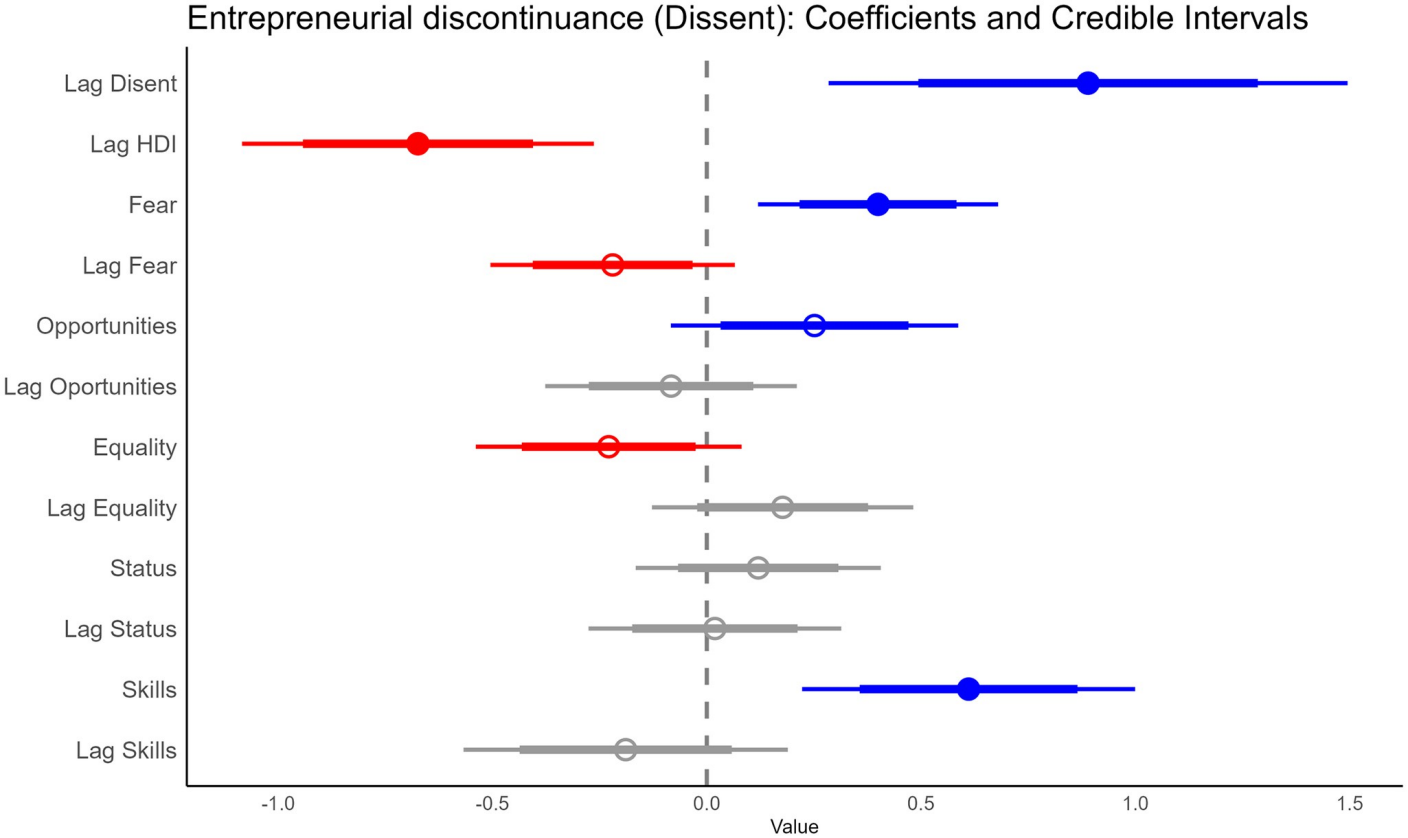
Coefficient plot for Dissent. It shows how business termination is related to its level in the previous year, to HDI, and to the proportion of people who fear failure, believe there are good opportunities to start a firm, desire equality in society, perceive entrepreneurs have high social status, and think they have skills to start a business in a given year. ‘Lag’ represents the previous year. Intercept is suppressed for simplicity. Thick lines represent 80% CI, while thin lines are 95% CI.

**Fig 6:**
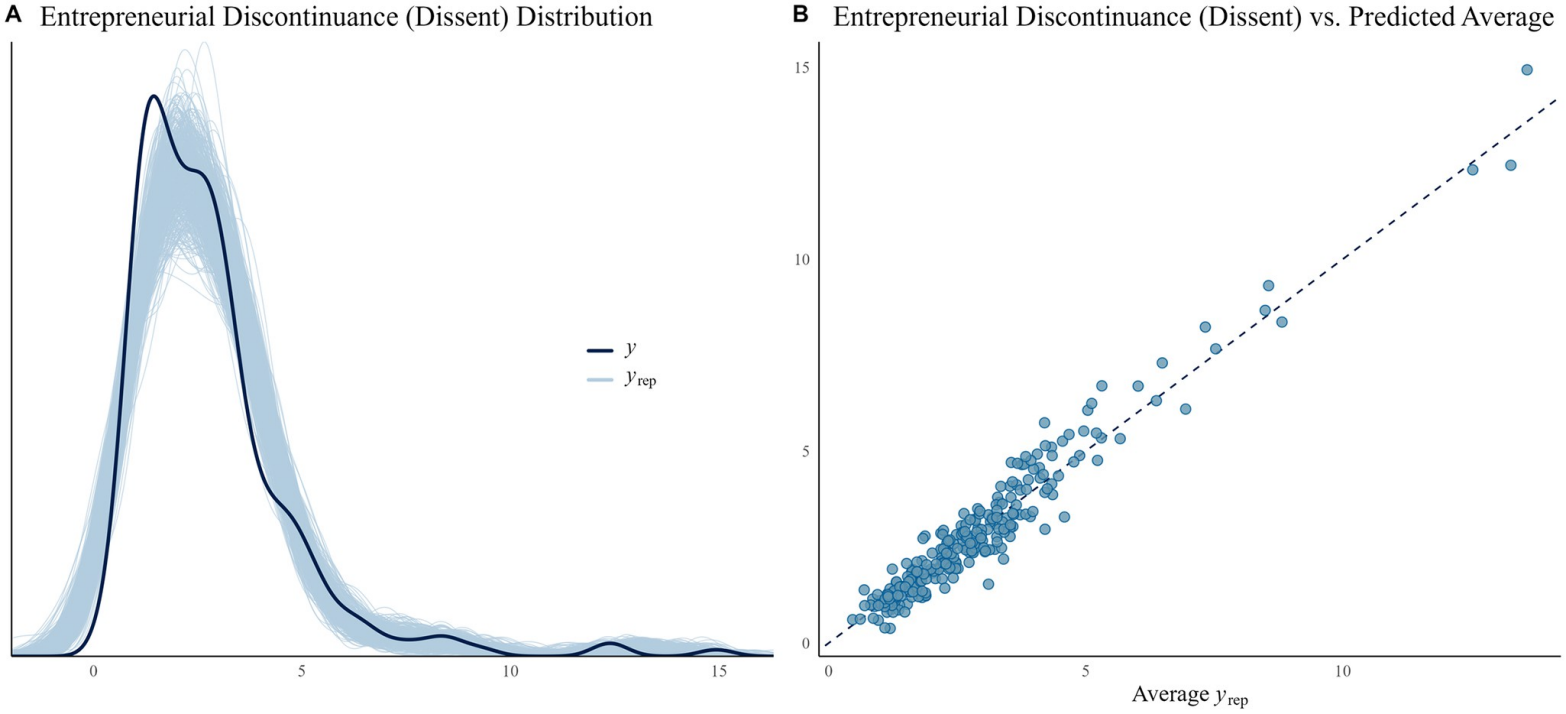
Model fitness of business exit (Dissent). A: Distribution (*y*) and random sample (500 randomly-selected replications) of predicted distributions (*y*_*rep*_). B: Average estimation of business exit vs. actual business exit.

The Bayesian model for business exit shows that the previous year’s business exit has a positive relation to ‘Dissent’ (over 95% of the distribution of the median is greater than zero), while HDI in the previous period has a negative relation to it (over 95% of the distribution of the median is less than zero). ‘Fear’, ‘Opportunities’, and ‘Skills’ show a positive influence on business exit (for opportunities 80%, while for fear and skills 95%, the CI of their estimated median distribution was greater than zero). ‘Lag Fear’ and ‘Equality’ were found to have a negative relation to business exit (the 80% CI of their estimated median distribution was less than zero; see Fig 5. Other covariates did not appear to be related to the exit of the business. Fig 6 shows how closely the model is able to replicate business exit; panel A shows the estimated distribution and the actual distribution of dissent, while panel B shows the average estimate versus the data.

Taking into account the results of all the models (Figs 1, 3 and 5), we can reject our working hypothesis that there is a positive association between human development and entrepreneurial activity. Models show a negative relationship between higher levels of human development (i.e., HDI lag) and the three stages of entrepreneurialization under study (intention, early, and exit). The random effects models used in the analysis indicate that there are intrinsic differences between countries and years that are taken into account in the estimation (see S1 Fig).

## Discussion

Our findings show that human development is negatively associated with entrepreneurial activity (the distribution of HDI lag coefficients is negative for the three models). This result implies that a decrease in a country’s living standards would be expected to be accompanied by a movement in the opposite direction in the rate of entrepreneurial activity. Similarly, an increase in the development conditions of a nation could be associated with lower rates of entrepreneurship. An explanation could be that improved economic opportunities reduce the need for entrepreneurship. Acs et al. [76] argued that as economies develop, the inclination toward entrepreneurship diminishes due to the growth of firm size due to improved managerial abilities, higher returns from work wages, and improvements in infrastructure that favor larger firms.

Given that our results indicate that entrepreneurship is a response to development conditions, we can frame our findings as entrepreneurship being used as a vehicle for economic mobility (i.e., in response to low levels of development) to overcome poverty and inequality [77]. This relates to the idea of necessity entrepreneurship, recently re-conceptualized by Dencker et al. [78], in which human beings—by the persecution of their own entrepreneurial endeavors—resort to their abilities to create and produce. Thus, it appears that people who observe a weak economic, health, and educational environment rely on their own capabilities to generate economic value. This insight also relates to the literature on the entrepreneurial ecosystem (EE), as we can argue that decreasing the levels of human development in a nation affects components of EE—as stated by Stam and van de Ven [79]—such as physical infrastructure, demand, and finance, but at the same time, it could give rise to better conditions in the remaining elements of EE, such as talent, knowledge, and leadership.

The results of our study contribute to the discussion of economic growth and entrepreneurship, suggesting that there may not be a triad of positive feedback between entrepreneurship, economic growth, and human development. If there is positive feedback, better socioeconomic conditions could help ongoing ventures thrive and become established businesses, since favorable variations of human development levels could foster markets. However, our results contradict this idea, suggesting that entrepreneurial activities could be more of an alternative to fixed income employment or other classical jobs, making the relationship between entrepreneurial activity and economic growth more nuanced [11].

In particular, the level of human development showed consistent negative associations in the three stages of entrepreneurship under study: early stage, intention, and discontinuation of ventures. This consistency strengthens the argument for a relationship between HDI and entrepreneurial activity.

For both the early and future stages, there appears to be a systemic effect that makes both stages have similar trends regarding human development. As the conditions of human development worsen, the early stages and intentions for future endeavors appear positively associated. However, entrepreneurial discontinuance (despite having a significant negative association) suggests a different effect: While environmental conditions improve, a positive trend toward entrepreneurial discontinuance is expected.

Our findings complement those previously stated by Amorós and Cristi [17], who found that human development has a U-shaped association with both TEA and necessity entrepreneurship, so for lower levels of development, there is a negative association between them. This negative association is similar to the research by Pisá-Bó et al. [80], who found that for the Spain regions between 2002 and 2014, the lack of GDP growth per capita was enough to promote entrepreneurship. However, our results also contrast with other research findings, which found a positive association between human development and entrepreneurial activity [24–26].

The results also depict the persistence of entrepreneurial activity over time, as the level of entrepreneurship in the preceding year is positively associated with the current year’s level across the three stages. However, the relationship is not as strong for entrepreneurial intentions as for the other stages. For early entrepreneurship and entrepreneurial discontinuation, the result may be interpreted as “stickiness” in entrepreneurial activity, perhaps due to inertia or macroeconomic trend lags.

Regarding aggregates of individual entrepreneurship determinants, we found that fear of failure positively relates to entrepreneurial discontinuation and early entrepreneurship. However, we did not find enough evidence that it relates to entrepreneurial intention. A potential explanation could be related to the complexity of fear as an emotion [60], as research focusing on risk, a concept related to fear of failure, has found inconsistent effects throughout studies [81]. Furthermore, it has been argued that entrepreneurial intention could be better explained by an individual’s ability to recognize opportunities than by their risk-bearing attitudes [82]. In particular, fear in the current period might generate venture discontinuations for the entrepreneurial discontinuance model. However, the fear rate in the previous period appears negative (albeit not for the whole distribution). Fear in the current period can be related to mistrust in macroeconomic conditions, so it could be reasonable to expect a positive association with the discontinuation of ventures. The relation to fear levels in the previous periods might be more complex and dynamic, perhaps with people involved in ventures becoming cautious of adverse macroeconomic conditions and taking active measures to diminish the likelihood of discontinuing their ventures. However, other dynamics could shape our results, which would require a more careful examination in future research.

Perception of opportunities is positively related to the three entrepreneurial stages under study. This result is in line with the previous literature finding a positive association between (perceived) opportunities in the business landscape and the intention of becoming an entrepreneur [38, 83, 84]. However, the positive relationship with the discontinuation of ventures could require a closer examination. We hypothesize that greater environmental opportunities could lead entrepreneurs to close their ventures and restart or reemerge in other industries.

For entrepreneurial intentions, opportunities from the previous year are negatively associated with them. It is possible that opportunities detected in the previous period have already materialized, driving the measure of intentions (Futsup) to decrease. An alternative explanation relates to data collection by GEM since the opportunity index is based on perceptions of people not currently involved in any stage of entrepreneurial activity. Therefore, the perception of opportunities might differ between those foreign to entrepreneurial activity and those who were active.

Regarding the status given in a country to successful entrepreneurs, we did not find significant statistical evidence in any of the three models. This result is similar to that of Fragoso et al. [85], who did not find that social recognition influences entrepreneurial intent. Assessment of skills in a country was consistently positively associated with the three stages of entrepreneurship. This finding is in line with other related literature that has found a positive link between skills and entrepreneurial intentions when looking at students from developed countries [86, 87], MBA graduates [88], or people interested in taking entrepreneurship courses [89]. For discontinuance, the positive relation with skill perception appears counterintuitive. A potential explanation—based on literature about re-emergence, learning from failure, and serial entrepreneurs—is that if people believe they are highly qualified to start a business, they could be shutting down previous ventures to start new ones [90–92].

Furthermore, our findings revealed that country and year specific factors influence the slope of the relationship between our variables of interest. This implies that the relationship between variables changes across different countries and changes over time. Random effects capture the unobserved heterogeneity at the country level, implying inherent differences between countries that endure over the years. Furthermore, we identified that the estimates of the covariates are also influenced by random effects at the country level, implying that their impact on the outcome variable differs across countries and, by considering them, we have taken into account the unique characteristics of each country that influence the estimated effects of the explanatory variables. In general, the described results contribute to the literature on the determinants of entrepreneurship at the country level. Previous research has found that the phenomenon is multifaceted and depends on variables of different nature, such as the level of uncertainty in the economic policy of the country [93], the fragility of the state [94], and institutional characteristics [95].

Overall, our findings have important implications for social and economic policy that need to take into account the multifaceted nature of entrepreneurship and its negative relation to a country’s development. An economy will need to design tailored policies if it wants to maintain entrepreneurship levels while growing, incorporating the increasing opportunity cost and risks of the endeavor, to avoid entrepreneurship to falter. Economies struggling with development should focus on stabilization and support policies for entrepreneurs, since they have appropriate conditions for inherent entrepreneurship to flourish, but entrepreneurs probably cannot recover from failure. Policies oriented to innovation should consider the negative relation development has on entrepreneurship; as the decrease in the last might dry out innovation and increase monopolistic power. To counteract this effect, countries should create stronger policies oriented to increase innovation and the speed of the business life cycle, creating a virtuous relationship, and contributing to increase entrepreneurship levels, drive innovation, and reduce monopolistic power.

Regarding the limitations of our work, we acknowledge the existence of other relevant macroeconomic variables that could affect entrepreneurial activity [7, 96–98]. However, explicit inclusion of these variables falls outside the scope of this article and is left for future studies. Furthermore, the HDI measure may only partially encompass the comprehensive effect of human development on entrepreneurship, as it could overlook other elements such as equality, safety, and diversity. Although the environmental factors in our study attempt to integrate these overlooked dimensions, they are not comprehensive and may not fully reflect social perceptions or capture the inherent motivational influences. Finally, despite being one of the most widely used indexes for human development, academic concerns have been raised about the HDI. It could be thought that our results are driven by the way in which the dimensions encompassed by the HDI are aggregated, since they are given an equal distribution of weights. For example, McGillvray et al. [99] call for weights that vary according to “achievements and countries.” However, this contrasts with the previous work by Stapleton and Garrod [100] who show that there is no strong evidence to require HDI to have different weights, as it adds complexity that ultimately counteracts fit goodness. This is, of course, a discussion that requires further attention, particularly of a mathematical and methodological nature. However, given the extensive use of HDI and its parsimony, we assume it as a limitation of our study in the hope of having a clearer understanding of a correct measure of human development in the future. To better understand the impact that different factors have on entrepreneurship, it is necessary to study further the nature of entrepreneurship (theoretically and empirically), while incorporating motivational aspects (necessity and opportunity [12, 76, 101]); as well as its impact on other social dimensions, such as distributional justice [102]. Addressing these research avenues in future academic work will increase our understanding of the nuanced interplay between human development and entrepreneurship.

## Conclusion

The relationship between economic growth and entrepreneurship has been largely conceptualized in the literature as symbiotic. However, few studies have looked at the impact of economic development—beyond GDP measurements—on entrepreneurship, despite the positive feedback argument that environmental conditions could pave the way to nurturing more business opportunities. We attempt to fill this gap by shifting the analysis from pure economic growth to consider a more holistic view of development—human development—as a predictor of entrepreneurial activity. Mainly, we studied whether a country’s human development level is associated with entrepreneurial activity in three stages: early entrepreneurship, the intention to engage in future entrepreneurial efforts, and the discontinuation of existing ventures.

Our findings contradict the positive feedback argument and reveal that human development is negatively associated with early entrepreneurship and the proportion of people considering undertaking future entrepreneurial ventures. This suggests that conditions of lower human development in a nation could spur higher levels of nascent entrepreneurial activity and an increased likelihood of future entrepreneurial activity. At the same time, we found that more deficient conditions of human development are associated with increased discontinuation of businesses. These results suggest a paradoxical relationship: areas with lower human development may inspire higher entrepreneurial start-ups and intent due to necessity, perhaps due to limited job opportunities or lower wage levels. However, the same environment appears to make sustaining these ventures more difficult, as evidenced by the higher discontinuation rate.

The results stimulate reflections on the nature and motivations of entrepreneurship, hinting at the distinction between opportunity and necessity entrepreneurship. This dichotomy separates those who enter entrepreneurship because they identify an opportunity from those who do so out of necessity, typically because of the absence of better options. Our findings also underscore the importance of supportive environments for the survival of new ventures. Although lower levels of human development might motivate entrepreneurial activity, they appear to simultaneously impose challenges that could compromise the longevity of these businesses. In light of these results, policy initiatives aimed at fostering entrepreneurship should consider measures to stimulate start-ups and strategies to improve the conditions for firm survival, particularly in regions with lower levels of human development. If improving human development seems to stifle entrepreneurial activity, policymakers must find a balance: to secure development efforts while preventing discouraging entrepreneurial ventures.

## Supporting information

S1 Fig Distribution of coefficients’ standard deviation by country (intercept and slope) and year (intercept) random effects. ‘l.Y’ represents the lag of the dependent variable for each model.
(TIF)
