# Supplementary figures and images for: Human development and entrepreneurship: A cross-country analysis of early-stage, intention, and discontinuation
Source: PLoS One. 2024 Nov 25;19(11):e0313678. doi: 10.1371/journal.pone.0313678 (PMC11588273; doi:10.1371/journal.pone.0313678)

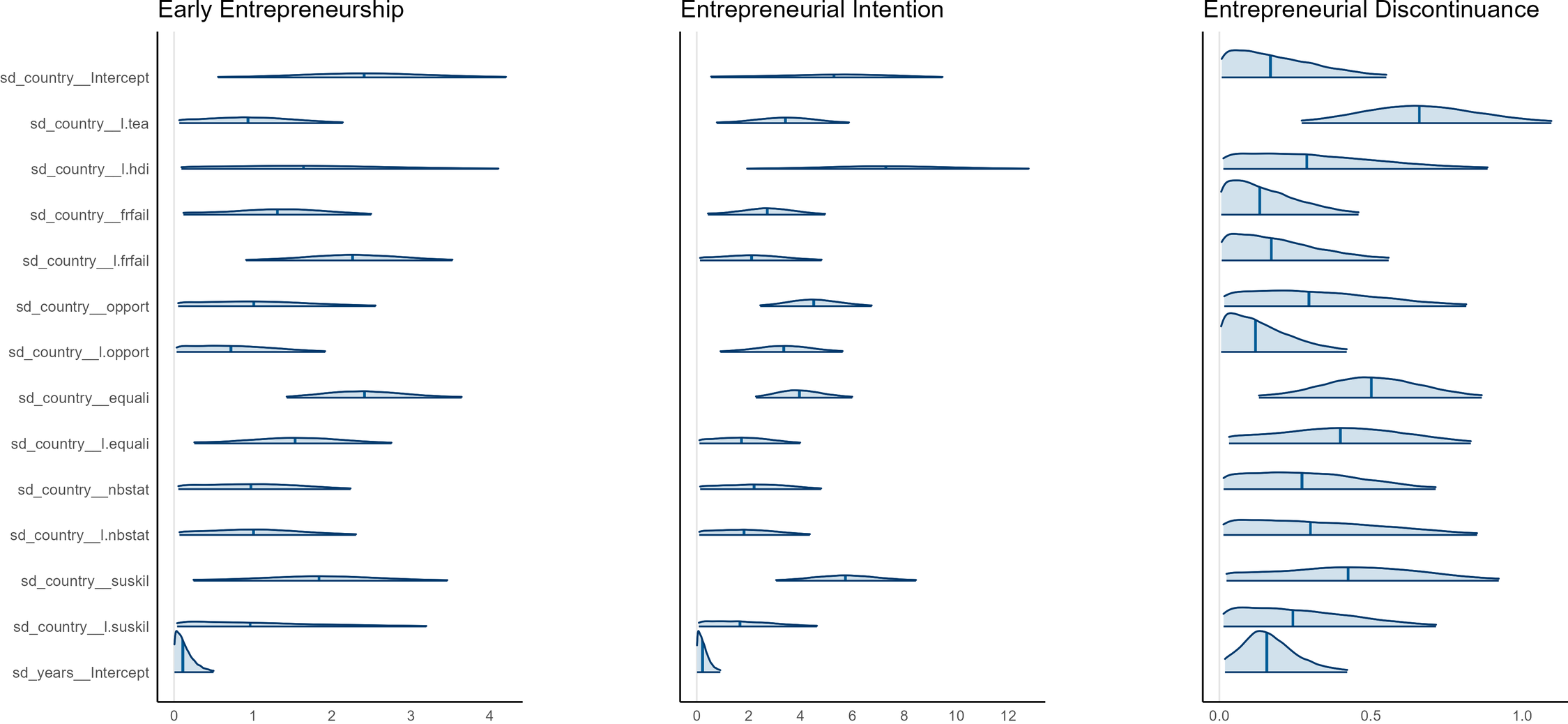

Supplement: S1 Fig — ‘l.Y’ represents the lag of the dependent variable for each model. (TIF) [file pone.0313678.s001.tif]
